# Is plasma amyloid-β 1–42/1–40 a better biomarker for Alzheimer’s disease than AβX–42/X–40?

**DOI:** 10.1186/s12987-022-00390-4

**Published:** 2022-12-03

**Authors:** Hans-Wolfgang Klafki, Barbara Morgado, Oliver Wirths, Olaf Jahn, Chris Bauer, Hermann Esselmann, Johannes Schuchhardt, Jens Wiltfang

**Affiliations:** 1 Department of Psychiatry and Psychotherapy, University Medical Center Goettingen, Georg-August-University, Von-Siebold-Str. 5, 37075 Goettingen, Germany; 2 Neuroproteomics Group, Department of Molecular Neurobiology, Max Planck Institute for Multidisciplinary Sciences, Hermann-Rein-Straße 3, 37075 Goettingen, Germany; 3 MicroDiscovery GmbH, Marienburger Strasse 1, 10405 Berlin, Germany; 4 German Center for Neurodegenerative Diseases (DZNE), Goettingen, Germany; 5 Neurosciences and Signaling Group, Institute of Biomedicine (iBiMED), Department of Medical Sciences, University of Aveiro, Aveiro, Portugal

**Keywords:** Alzheimer’s disease, Biomarker, Amyloid-β peptides, Blood plasma, Aβ42/40 ratio, Immunoassay

## Abstract

**Background:**

A reduced amyloid-β (Aβ)42/40 peptide ratio in blood plasma represents a peripheral biomarker of the cerebral amyloid pathology observed in Alzheimer’s disease brains. The magnitude of the measurable effect in plasma is smaller than in cerebrospinal fluid, presumably due to dilution by Aβ peptides originating from peripheral sources. We hypothesized that the observable effect in plasma can be accentuated to some extent by specifically measuring Aβ1–42 and Aβ1–40 instead of AβX–42 and AβX–40.

**Methods:**

We assessed the plasma AβX–42/X–40 and Aβ1–42/1–40 ratios in an idealized clinical sample by semi-automated Aβ immunoprecipitation followed by closely related sandwich immunoassays. The amyloid-positive and amyloid-negative groups (dichotomized according to Aβ42/40 in cerebrospinal fluid) were compared regarding the median difference, mean difference, standardized effect size (Cohen’s d) and receiver operating characteristic curves. For statistical evaluation, we applied bootstrapping.

**Results:**

The median Aβ1–42/1–40 ratio was 20.86% lower in amyloid-positive subjects than in the amyloid-negative group, while the median AβX–42/X–40 ratio was only 15.56% lower. The relative mean difference between amyloid-positive and amyloid-negative subjects was −18.34% for plasma Aβ1–42/1–40 compared to −15.50% for AβX–42/X–40. Cohen’s d was 1.73 for Aβ1–42/1–40 and 1.48 for plasma AβX–42/X–40. Unadjusted p-values < 0.05 were obtained after .﻿﻿632 bootstrapping for all three parameters. Receiver operating characteristic analysis indicated very similar areas under the curves for plasma Aβ1–42/1–40 and AβX–42/X–40.

**Conclusions:**

Our findings support the hypothesis that the relatively small difference in the plasma Aβ42/40 ratio between subjects with and without evidence of brain amyloidosis can be accentuated by specifically measuring Aβ1–42/1–40 instead of AβX–42/X–40. A simplified theoretical model explaining this observation is presented.

**Supplementary Information:**

The online version contains supplementary material available at 10.1186/s12987-022-00390-4.

## Background

The amyloid-β (Aβ)42/40 ratio in blood plasma has turned out to represent a highly attractive and robust peripheral biomarker of the cerebral amyloid pathology associated with Alzheimer’s disease (AD) [1–5]. Furthermore, a number of recent studies have shown that also the plasma concentrations of specific phosphorylated forms of tau protein, namely p-tau181, ptau217 and ptau231, can reliably detect an abnormal brain Aβ status as indicated by Aβ-positron emission tomography (Aβ-PET) or low CSF Aβ42/40 [6–11]. Recently, the first plasma Aβ42/40 assay gained approval by the Centers for Medicare & Medicaid Services under the Clinical Laboratory Improvement Amendments (CLIA) protocol [12]. This assay is based on Aβ immunoprecipitation followed by enzymatic cleavage and quantification of specific proteolytic carboxy-terminal Aβ fragments by liquid chromatography–mass spectrometry [13]. A major concern regarding the applicability of the plasma Aβ42/40 ratio to routine use is the rather modest decrease in those subjects with evidence of brain amyloid pathology (amyloid-positive subjects) compared to amyloid-negative individuals. While the Aβ42/40 ratio in cerebrospinal fluid (CSF) was reported to be approximately 50% lower in the presence of amyloid [14], the observed magnitude of the group differences in plasma Aβ42/40 between amyloid-positive and amyloid-negative subjects was approximately 10–15% [1, 2]. In good agreement with these reports we observed a 14% lower mean plasma Aβ42/40 ratio in patients with dementia due to AD compared to patients with dementia due to other reasons in a previous study employing a “two-step immunoassay” [15]. It is clear that for routine use extremely robust and precise plasma Aβ42/40 assays will be required.

The pool of circulating Aβ in blood comprises Aβ peptides originating from the central nervous system (CNS) as well as Aβ peptides originating from peripheral sources [1, 16]. A number of studies employing Aβ-immunoaffinity chromatography or Aβ-immunoprecipitation followed by mass spectrometry (IP-MS) have indicated that the majority of soluble Aβ peptides in human CSF carries a free N-terminal aspartic acid residue in position one of the Aβ amino acid sequence [Asp(1)] [17–24]. In sharp contrast, 2D-Western blot analysis and IP-MS indicated that blood plasma contains appreciable amounts of Aβ variants with amino-termini other than Asp(1) [25, 26].

We hypothesized that the decrease in plasma Aβ42/40 in the presence of brain amyloid pathology reflects a pool of highly soluble Aβ in the CNS and propose that the measurable magnitude of this decrease in plasma may possibly be accentuated by excluding at least some of the plasma Aβ originating from peripheral sources. Theoretically, this might be achievable by employing Aβ assays which are specific for Aβ1–42 and Aβ1–40 instead of assays detecting also Aβ variants with other N-termini than Asp(1). Herein, the Aβ variants with an unspecified N-terminus are referred to as AβX-42 and AβX-40, respectively. This way, some of the N-terminally modified Aβ variants originating from the periphery will not be detected, resulting in a net increase in the relative contribution of Aβ peptides originating from the CNS to the Aβ signals measured in blood plasma.

Here, we set out to test this hypothesis by investigating the ratios AβX–42/AβX–40 detected by monoclonal antibody (mAb) 6E10 and Aβ1–42/Aβ1–40 detected by mAb 3D6 in plasma and CSF side-by-side in a carefully preselected clinical sample dichotomized by an unbiased and purely neurochemical approach.

## Materials and methods

### Study cohort and study approval

The study was conducted according to the revised Declaration of Helsinki and good clinical practice guidelines. All study participants were recruited at the Department of Psychiatry and Psychotherapy at the University Medical Center Goettingen. The ethics committee of the University Goettingen approved the pseudonymized collection of biological samples and clinical data in the local biobank and their use in biomarker studies (9/2/16). Written informed consent was obtained from all subjects or their legal representatives prior to inclusion. The study cohort was pre-selected from the local biobank and comprised originally 78 subjects for whom CSF and EDTA-blood plasma samples were available. All of the study participants were part of a previous study [27]. According to a biomarker-supported clinical diagnosis, 40 of the study participants included here were diagnosed as having improbable Alzheimer’s disease (AD) and 38 as having probable or possible AD. This clinical classification was based on clinical observations, CSF biomarkers (Aβ42/40 ratio, phospho-Tau181, total-Tau, measured in a clinical laboratory) and, whenever available, psychometric and neuroimaging biomarker data [27].

### Capillary isoelectric focusing immunoassay

For characterization of anti-Aβ monoclonal antibodies (mAbs) regarding their ability to recognize different N-terminal Aβ variants, we employed an automated Capillary Isoelectric Focusing (CIEF) Immunoassay on a Peggy-Sue instrument (Protein Simple, San Jose, California, 95134 USA) as described previously [28, 29]. In brief, synthetic Aβ or Aβ-related peptides were separated in microcapillaries by isoelectric focusing, immobilized photochemically to the inner capillary surface and probed with anti-Aβ mAbs in combination with a peroxidase labeled secondary antibody. The synthetic peptides Aβ1–38, Aβ1–40, Aβ1–42, Aβ2–40, Aβ3–40, AβN3pE–40 (pyroglutamate Aβ3–40), Aβ4–40, Aβ5–40 and Aβ11–40 were obtained from AnaSpec Inc., Fremont CA 94555, USA). The Aβ-related peptide Aβ−3–40 (APP669–711) [30] was kindly provided by Professor H.-J. Knölker, Technische Universität Dresden, Germany). The Aβ-related model peptide Aβ−﻿23–16 (APP649–687, H_2_N-GLTTRPGSGLTNIKTEEISEVKMDAEFRHDSGYEVHHQK-CONH_2_) was introduced previously [31]. The peptides were loaded as a mixture (Aβ1–40, Aβ2–40 and Aβ5–40) or individually. AβN3pE–40 was loaded at a concentration of 200 ng/mL while all other Aβ variants were loaded at a concentration of 100 ng/mL. MAb 1E8 was obtained from nanoTools, Teningen, Germany), mAb 6E10 from BioLegend (www.biolegend.com) and mAb 3D6 from Creative Biolabs, Shirley, NY 11967, USA (PABL-Cat. No. 011, anti Aβ1–5). MAb 4G8 can be purchased from BioLegend (www.biolegend.com) (previously Covance catalog# SIG-39200).

### Immunoprecipitation-mass spectrometry

In order to assess which N-terminal Aβ variants are immunoprecipitated by mAb 1E8 under similar conditions as used in the two-step immunoassay (see below), a mixture of synthetic Aβ peptides was prepared in Diluent-35 (Meso Scale Discovery (MSD), Rockville, MD, USA) and subjected to IP-MS. The mixture comprised Aβ1–38, Aβ1–40, Aβ1–42, Aβ2–40, Aβ3–40, AβN3pE–40, Aβ4–40, Aβ5–40, Aβ11–40, Aβ−3–40 (APP669–711) and the model peptide “Aβ−23–16”, each peptide at a concentration of 91 ng/mL. 27.5 µL of this peptide mixture was combined with 172.5 µL of Diluent-35, 200 µL of H_2_O and 100 µL of 5 × IP-buffer concentrate containing 250 mM HEPES/NaOH, pH 7.4, 750 mM NaCl, 2.5% Igepal CA630, 1.25% sodium deoxycholate; 0.25% SDS and Complete Mini Protease inhibitor cocktail (Roche Diagnostics GmbH, Mannheim, Germany, 1 tablet per 2 mL). After addition of 15 µL of mAb 1E8 coupled and crosslinked to Dynabeads M-280 Sheep anti-Mouse IgG (Invitrogen/ThermoFisher Scientific, Waltham, MA, USA), the mixture was incubated for approximately 16 h on a mixer at 1400 RPM in a cold room at approx. 4–8 °C. The unbound material was removed and discarded, and the magnetic bead immune complexes were washed 3 × 5 min with phosphate buffered saline (PBS) containing 0.1% bovine serum albumin and 1 × 3 min with 10 mM Tris/HCl, pH 7.5. The beads were resuspended in 0.5 mL 50 mM ammonium acetate (pH ~ 7.0) and washed one more time with 50 mM ammonium acetate and 1 × with H_2_O. Finally, the bound Aβ peptides were eluted in 2.5 µL of 70% acetonitrile containing 5 mM HCl and analysed by matrix-assisted laser desorption mass spectrometry (MALDI-TOF-MS) as recently described in detail [24]. Briefly, the eluates (0.5 µL) were spotted onto a pre-structured MALDI sample support (MTP AnchorChip 384 BC; Cat. No. 8280790, Bruker, Bremen, Germany), followed by the addition of 0.5 µL matrix solution consisting of 5 mg/mL 2-cyano-4-hydroxycinnamic acid (CHCA, Cat. No. 709905-1G, Sigma Aldrich/Merck, Taufkirchen, Germany) in 50% acetonitrile/0.05% trifluoroacetic acid. A 1:1 mixture of Peptide Calibration Standard II (Cat. No. 8222570, Bruker, Bremen, Germany) and PepMix2 (Cat. No. C102, LaserBio Labs, Valbonne, France) was used as calibrant. The samples were dried and positively charged ions in the m/z range of 1800–6000 were recorded in the reflector mode using an ultrafleXtreme MALDI-TOF/TOF mass spectrometer operated under the software flexControl 3.4 (Bruker, Bremen, Germany). A total of 5000 spectra per sample were recorded from different spot positions and the software flexAnalysis 3.4 (Bruker, Bremen, Germany) was used to annotate and calibrate monoisotopic masses with the implemented SNAP2 algorithm and cubic calibration.

### SULFO-TAG labeling of monoclonal antibody 3D6

The mAb 3D6 (Creative Biolabs, PABL-Cat. No. 011, anti Aβ1-5) was labeled with SULFO-TAG according to the instructions provided with the MSD Gold SULFO-TAG NHS-Ester Conjugation Pack 1 (Mesoscale Discovery (MSD), Rockville, MD, USA, Cat. No R31AA1). In brief, 8.9 µL of a freshly prepared 3 nmol/µL solution of MSD Gold SULFO-TAG NHS-Ester was carefully added dropwise to 200 µL of a 1.0 mg/mL solution of mAb 3D6 in phosphate buffered saline (PBS). After careful mixing by pipetting the solution up and down several times, the reaction was incubated in the dark for 2 h at room temperature. The remaining unbound SULFO-TAG was removed by buffer exchange into conjugate storage buffer (PBS, pH 7.4 containing 0.05% sodium azide) on a Zeba Spin desalting column (40 K MWCO, 0.5 mL). The SULFO-TAG labeled 3D6 antibody was stored at 4 °C in the dark until use.

### Semi-automated Aβ-immunoprecipitation

Aβ-peptides were immunoprecipitated from EDTA-blood plasma samples in a semi-automated fashion using the CyBio FeliX liquid handling instrument (Analytik Jena, Jena, Germany) following a modified version of our previously published IP-protocol [31]. Aliquots of EDTA-blood plasma samples (approximately 500 µl, each) were stored at –80 °C in Matrix 0.5 mL tubes (Thermo Scientific). All samples were thawed at room temperature and mixed vigorously for 5 × 10 s. Insoluble material was removed by centrifuging the tubes within a Matrix Rack for 10 min at 4350 × g in a swing-out rotor. The rack containing the centrifuged samples was placed inside the CyBio FeliX instrument, which transferred 250 µL of each supernatant into a 1.2 mL polypropylene 96 deep well plate (MegaBlock (Sarstedt, Germany). From there, 200 µL of each EDTA-plasma sample were pipetted into a separate 96 deep well plate and mixed with 200 µL of H_2_O, 100 µL of 5 × IP-buffer concentrate (250 mM HEPES/NaOH, pH 7.4, 750 mM NaCl, 2.5% Igepal CA630, 1.25% sodium deoxycholate; 0.25% SDS and Complete Mini Protease inhibitor cocktail (Roche Diagnostics GmbH, Mannheim, Germany, 1 tablet per 2 mL)) and 25 µL of functionalized 1E8 magnetic beads (see above). After overnight incubation at 4 °C with continuous agitation at 1000 RPM on an Eppendorf ThermoMixer C (Eppendorf, Hamburg, Germany), the MegaBlock was relocated to the CyBio Felix instrument for automated washing and elution. For the immobilization of the magnetic bead immune complexes an ALPAQUA MAGNUM FLX Universal Magnet (Beverly, MA, USA) adapter was used. The supernatants were discarded, and the magnetic beads were washed 3 × for 5 min with 1 mL of PBS/0.1% BSA and 1 × for 3 min with 1 mL of 10 mM Tris/HCl, pH 7.5. Per well, 35 µL of PBS containing 0.05% Tween-20 (PBS-T) were added, and the Aβ peptides were eluted from the magnetic bead immune complexes by heating the 96 well round bottom deep well plate without a lid for 5 min at a set temperature of 99 °C and 1100 RPM in a BioShake 3000-T elm Deep Well (QInstruments, Germany). A remaining volume of approximately 20 µL of IP-eluate per sample was obtained. The eluates and magnetic beads were transferred into to a 500 µL Protein LoBind 96 deep well Plate (Eppendorf, Hamburg, Germany) and diluted fourfold by adding 60 µL of Diluent-35 (MSD). After immobilization of the beads using the magnet adaptor, the diluted bead-free eluate was transferred to a fresh LoBind 96 deep well plate. Finally, the diluted IP-eluates were divided into two aliquots, pipetted into Matrix 0.5 mL tubes and stored at −80 °C until the measurements on Aβ multiplex immunoassays.

### Quantification of Aβ isoforms by MSD multiplex immunoassays

The concentrations of AβX–40 and AβX–42 in CSF and fourfold diluted IP-eluates from plasma were determined with the commercially available MSD Aβ panel 1 (6E10) V-PLEX multiplex assay kit (Meso Scale Discovery (MSD), Rockville, MD, USA). For measuring Aβ1–40 and Aβ1–42 instead, the 6E10-sulfotag detection antibody was replaced by 3D6-sulfotag. CSF samples were measured after 16-fold dilution with Diluent-35 (MSD) [32]. For the measurements of AβX–40, AβX–42, Aβ1–40 and Aβ1–42 in fourfold diluted IP-eluates from plasma, the assay protocol was modified, slightly: Following the kit instructions, the assay plate was blocked with 150 µL of Diluent-35 per well for 1 h at room temperature with constant agitation followed by 3 washing steps with 150 µL of PBS-T per well. Then, 15 µl of fourfold diluted IP-eluate or calibrator dilution plus 15 µL of the 6E10-sulfotag or 3D6-sulfotag detection antibody dilution were pipetted into each well (final reaction volume: 30 µL per well). After 2 h incubation at room temperature on a mixer and 3 × washing with PBS-T, 150 µL of 2 × Read Buffer was added to each well and the plate was immediately read on a MSD QuickPlex SQ 120 reader (MSD). All assays were performed with two technical replicates of each sample on the same assay plate.

### Statistics

All statistical evaluations were performed with R version 3.5.1. Baseline statistics is reported as mean ± standard deviations (Table 1). For comparing the measured Aβ isoform levels and Aβ42/40 ratios in CSF and diluted IP-eluates from blood plasma between amyloid-positive and amyloid-negative groups, we furthermore calculated medians and median absolute deviations with scaling factor 1.4826 (MAD) and used two-tailed Mann–Whitney tests. Scatterplots are shown on logarithmic scale, and correlation coefficients were calculated after logarithmic (log2) transformation. For the calculation of correlation coefficients, we used Pearson correlations. For fitting regression lines, we used a Deming regression (R package MethComp version 1.22.2), since both variables were measured experimentally.

**Table 1:** Characteristics of the study cohort

	All (n = 73)^a^	Aβ^−^ (n = 37)^b^	Aβ^+^ (n = 36)^b^	P-value^c^
Age [mean ± SD]	69.3 ± 7.8	67.1 ± 7.8	71.6 ± 7.2	0.0126
Female	42 (57.5%)	20 (54.1%)	22 (61.1%)	
Male	31 (42.5%)	17 (45.9%)	14 (38.9%)	
ApoE4 carrier	35 (47.9%)	8 (21.6%)	27 (75%)	
CSF AβX–42/X–40 [mean ± SD]	0.058 ± 0.025	0.082 ± 0.0055	0.033 ± 0.0056	< 0.0001
CSF AβX–42 [pg/mL, mean ± SD]	380.8 ± 217.8	530.0 ± 198.4	227.4 ± 96.7	< 0.0001
CSF AβX–40 [pg/mL, mean ± SD]	6678.2 ± 2598.4	6377.3 ± 2132.2	6987.4 ± 3003.2	0.5067
CSF t-Tau CSF [pg/mL, mean ± SD]^d^	417.5 ± 344.6	225.6 ± 80.2	614.8 ± 398.5	< 0.0001
CSF pTau181 [pg/mL, mean ± SD]^d, e^	60.0 ± 33.7	39.5 ± 11.3	81.0 ± 36.0	< 0.0001

*Aβ* amyloid-β

^a^Five subjects (out of originally 78) were excluded from the statistical analysis resulting in a final sample size of n = 73

^b^The clinical sample was dichotomized according to the AβX–42/X–40 ratio in cerebrospinal fluid (CSF) measured with the MSD Aβ panel 1 (6E10) V-PLEX multiplex assay

^c^Two-tailed Mann–Whitney test p-values for the comparison between the groups amyloid-negative (Aβ^−^, CSF AβX–42/X–40 > 0.058, n = 37) and amyloid-positive (Aβ^+^, CSF AβX–42/X–40 ≤ 0.058, n = 36)

^d^CSF levels of total Tau (t-Tau) and phospho-Tau-181 (pTau181) were routinely determined in a clinical laboratory

^e^For one subject, the measured pTau181 concentration was < 15.6 pg/mL. For the statistical analysis, this value was artificially set to a fixed value of 15.6 pg/mL

For comparing the AβX–42/X–40 and Aβ1–42/1–40 ratios in plasma and assessing the impact of replacing the mAb 6E10 by mAb 3D6 on the detection of amyloid-positivity, we used three different parameters as measures of the magnitude of the effect:

- (i) The relative median difference, calculated as:

$$Median \,difference \left( \% \right) = 100*\frac{{median \left( {A\beta^{ + } } \right) - median \left( {A\beta^{ - } } \right)}}{{median\left( {A\beta^{ - } } \right)}}$$

- (ii) The relative mean difference, calculated as:
  $$Mean\,difference \left( \% \right) = 100*\frac{{mean \left( {A\beta^{ + } } \right) - mean \left( {A\beta^{ - } } \right)}}{{mean\left( {A\beta^{ - } } \right)}}$$

- (iii) Cohen’s d (standardized effect size), calculated with R package “effsize” (version 0.8.1).

For testing the significance of the observed difference of effect sizes we applied a .632 bootstrapping (re-sampling of patients with replacement including refinement of the estimator as proposed by Efron in 1983 [33]. We applied 1000 replications of the bootstrapping and calculated the difference of effect sizes (e.g. 0.632 × median difference of the resampling﻿ + 0.368 × median difference of data without resampling). The differences of the resulting effect sizes are normally distributed (Shapiro p-value: 0.85). Making use of this fitted normal distribution, bootstrapping p-values were calculated by using a normal distribution after normalization of the standard deviation.

Single value ROC curves were calculated with R-package pROC (version 1.18.0).

## Results

### Antibody selectivity

**Fig. 1:**
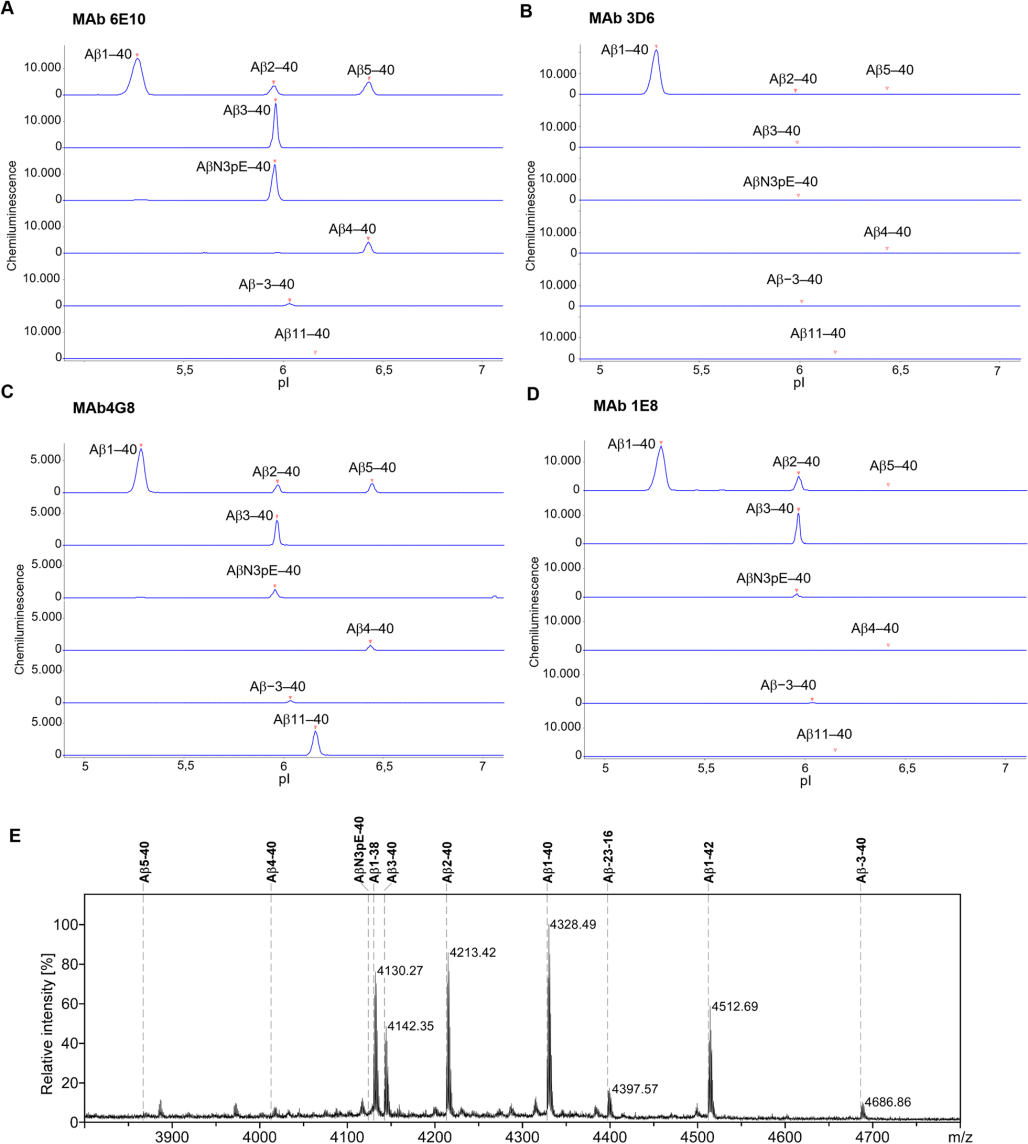
Assessment of antibody selectivity. **A**–**D** A series of synthetic N-terminal amyloid-β (Aβ) variants was separated by isoelectric focusing in microcapillaries, immobilized photochemically to the inner capillary wall and probed with the indicated monoclonal anti-Aβ antibodies. The peptides were loaded as a mixture or individually, as indicated. **A** Monoclonal antibody (mAb) 6E10 recognized Aβ40 variants starting at Asp(1), Ala(2), Glu(3) pyro-Glu(3), Phe(4), Arg(5) and Val(−3). Aβ11–40 was not detected. **B** MAb 3D6 strongly detected Aβ1–40, but essentially none of the other tested N-terminal Aβ variants. **C** MAb 4G8 recognized all of the tested Aβ variants. **D** MAb 1E8 recognized Aβ1–40, Aβ2–40, Aβ3–40 and AβN3pE–40. In addition, the N-terminally elongated Aβ−3–40 was recognized, albeit with a comparatively small signal. Aβ4–40, Aβ5–40 and Aβ11–40 were not detected. **E** MALDI-TOF-MS mass spectrum of Aβ peptide variants immunoprecipitated by monoclonal antibody 1E8. A mixture of synthetic Aβ peptides and Aβ related peptides was subjected to magnetic bead immunoprecipitation with mAb 1E8 followed by matrix-assisted laser desorption/ionization time-of-flight mass spectrometry (MALDI-TOF-MS) in reflector mode. The starting material included synthetic peptides corresponding to Aβ1–38 (calculated monoisotopic mass [M + H]^+^_mono, calc_ = 4130.019), Aβ1–40 (4328.156), Aβ1–42 (4512.277), Aβ2–40 (4213.129), Aβ3–40 (4142.092), AβN3pE–40 (4124.081), Aβ4–40 (4013.049), Aβ5–40 (3865.981), Aβ11–40 (3150.677), Aβ−3–40 (APP669–711; 4686.360) and the model peptide Aβ−23–16 (APP649–687; 4396.174). Under the experimental conditions, mAb 1E8 immunoprecipitated Aβ1–38, Aβ1–40, Aβ1–42, Aβ2–40, Aβ3–40 and the N-terminally elongated Aβ-related peptides Aβ−3–40 and Aβ−23–16 as indicated by the observed monoisotopic masses annotated in the mass spectrum. Aβ, amyloid-β; AβN3pE–40, Aβ40 peptide carrying an N-terminal cyclized pyroglutamic acid residue [pyro Glu(3)] in position 3 of the canonical Aβ amino acid sequence

The selectivity of mAbs directed against N-terminal Aβ epitopes and employed in this study was investigated by a CIEF immunoassay (Fig. 1). In brief, synthetic Aβ peptides were subjected to isoelectric focusing in microcapillaries followed by photochemical immobilization to the inner capillary wall and subsequent immunological detection. MAb 6E10, which serves as the detection antibody in the MSD Aβ panel 1 (6E10) assay kit, recognized synthetic peptides corresponding to Aβ1–40, Aβ2–40, Aβ3–40, AβN3pE–40, Aβ4–40 and Aβ5–40. Additionally, the N-terminally elongated Aβ−3–40 (APP669–711) was detected, albeit with a comparatively small signal (Fig. 1A), confirming previously published data [31]. Aβ11–40 was not detected. MAb 3D6, which serves for detection in the modified Aβ1–40 and Aβ1–42 MSD multiplex assay, showed excellent preference for Aβ carrying the free N-terminal Asp(1) (Fig. 1B). None of the tested N-terminally truncated or elongated Aβ variants produced an appreciable signal with mAb 3D6 under these conditions. To confirm that all tested Aβ variants are detectable in the CIEF immunoassay, mAb 4G8 was used as positive control. This pan-specific anti Aβ antibody is directed against Aβ17–24 and recognized all peptides, as expected, though with varying signal strength (Fig. 1C). Finally, mAb 1E8 was also included in the assessment as it is used for immunoprecipitation of Aβ from plasma prior to the MSD-measurements in the “two-step immunoassay”. MAb 1E8 detected Aβ1–40, Aβ2–40, Aβ3–40, AβN3pE–40 and, weakly, Aβ−3–40 (Fig. 1D). For mAb1E8 we additionally assessed the selectivity in magnetic bead immunoprecipitation (IP) (Fig. 1E). From a mixture of synthetic Aβ peptide variants, mAb 1E8 immunoprecipitated Aβ1–40, Aβ1–38, Aβ2–40, Aβ3–40 as well as N-terminally elongated Aβ−3–40 and Aβ−23–16. The peptides AβN3pE–40, Aβ4–40, Aβ5–40 and Aβ11–40 were essentially not recognized by mAb 1E8 under the tested IP conditions. Among the tested Aβ variants, Aβ−3–40 (a.k.a. APP669711) is of particular interest in the context of fluid biomarker research: The plasma Aβ−3–40/Aβ1–42 ratio (i.e. APP669–711/Aβ1–42 ratio) was shown to detect amyloid-positivity with high performance, and thus represents a further reliable plasma-based biomarker for Alzheimer’s disease [34, 35].

### Study cohort, data distribution and unbiased dichotomization of the sample

The pre-selected study cohort comprised 78 subjects for whom CSF and EDTA-blood plasma samples were available. The CSF-concentrations of AβX–40, AβX–42, Aβ1–40 and Aβ1–42 were measured with MSD multiplex assays employing mAb 6E10 or mAb 3D6 as detection antibodies, respectively. The corresponding EDTA-blood plasma samples were analyzed by Aβ immunoprecipitation followed by quantification of AβX–40, AβX–42, Aβ1–40 and Aβ1–42 with MSD multiplex assays in the IP-eluates (“two-step immunoassay”). The measured concentrations in diluted IP-eluates have to be considered relative plasma Aβ concentrations that may differ from the true plasma concentrations. As a first step in the data analysis, we checked the technical variance of the Aβ measurements of each sample on the MSD-immunoassays. Four subjects were excluded from all further statistical analyses because the coefficient of variation (CV) of the calculated Aβ concentrations was > 20% between duplicate reads for at least one of the analytes and in at least one of the assay runs. One additional study participant was excluded, because only singular data points were available for plasma AβX–40 and AβX–42 (due to a pipetting error). Thus, we continued the statistical analysis with a total sample size of n = 73.

**Fig. 2:**
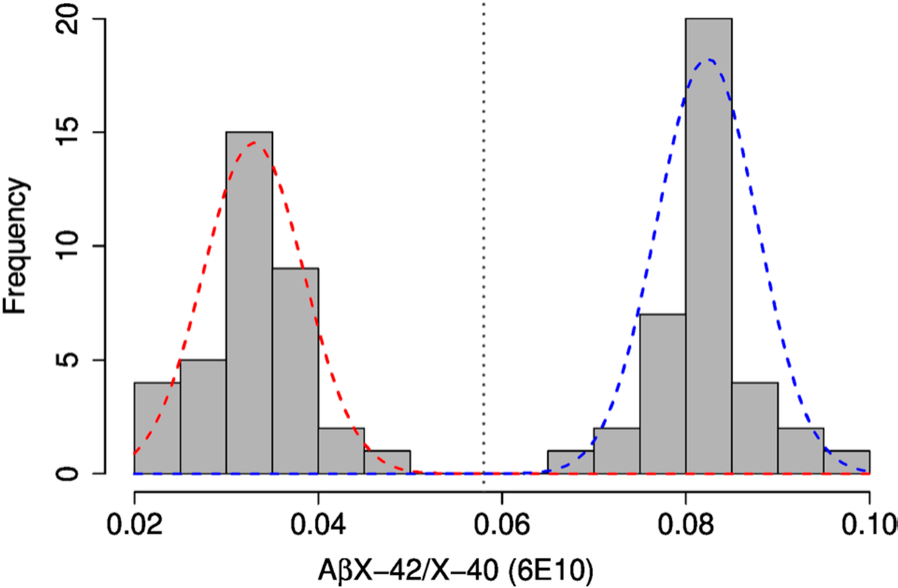
Distribution of the AβX–42/X–40 ratio in cerebrospinal fluid. The histogram shows the distribution of the AβX–42/X–40 ratios measured in cerebrospinal fluid (CSF) using the SULFO-TAG-6E10 detection antibody. Two normal distributions were fitted with the R package “mix-tools” (version 1.2.0). The vertical dashed line shows the intersection of the two curves at a CSF AβX–42/X–40 ratio of 0.058 (threshold). Aβ, amyloid-β

For an unbiased neurochemical dichotomization of the sample into the amyloid-negative and amyloid-positive subgroups, we chose the CSF AβX–42/X–40 ratio calculated from the CSF Aβ measurements with mAb 6E10. We observed a clear bimodal distribution of the CSF AβX–42/X–40 ratio. Two normal distributions with an intersection at an AβX–42/X–40 ratio of 0.058 could be fitted using a mixed model approach (Fig. 2).

Accordingly, the study participants were classified into the subgroups amyloid-positive (CSF AβX–42/X–40 ≤ 0.058, n = 36) and amyloid-negative (CSF AβX–42/X–40 > 0.058, n = 37). This neurochemical classification of the study participants was in good agreement with the above-mentioned biomarker-supported clinical diagnosis: All of the 37 amyloid-negative cases had been previously diagnosed as improbable AD. Of the 36 amyloid-positive cases, 35 had been previously diagnosed probable or possible AD and one as improbable AD. The characteristics of the final study cohort included in the statistical analysis (n = 73) are summarized in Table 1.

### Correlations between Aβ measurements with two different detection antibodies

**Fig. 3:**
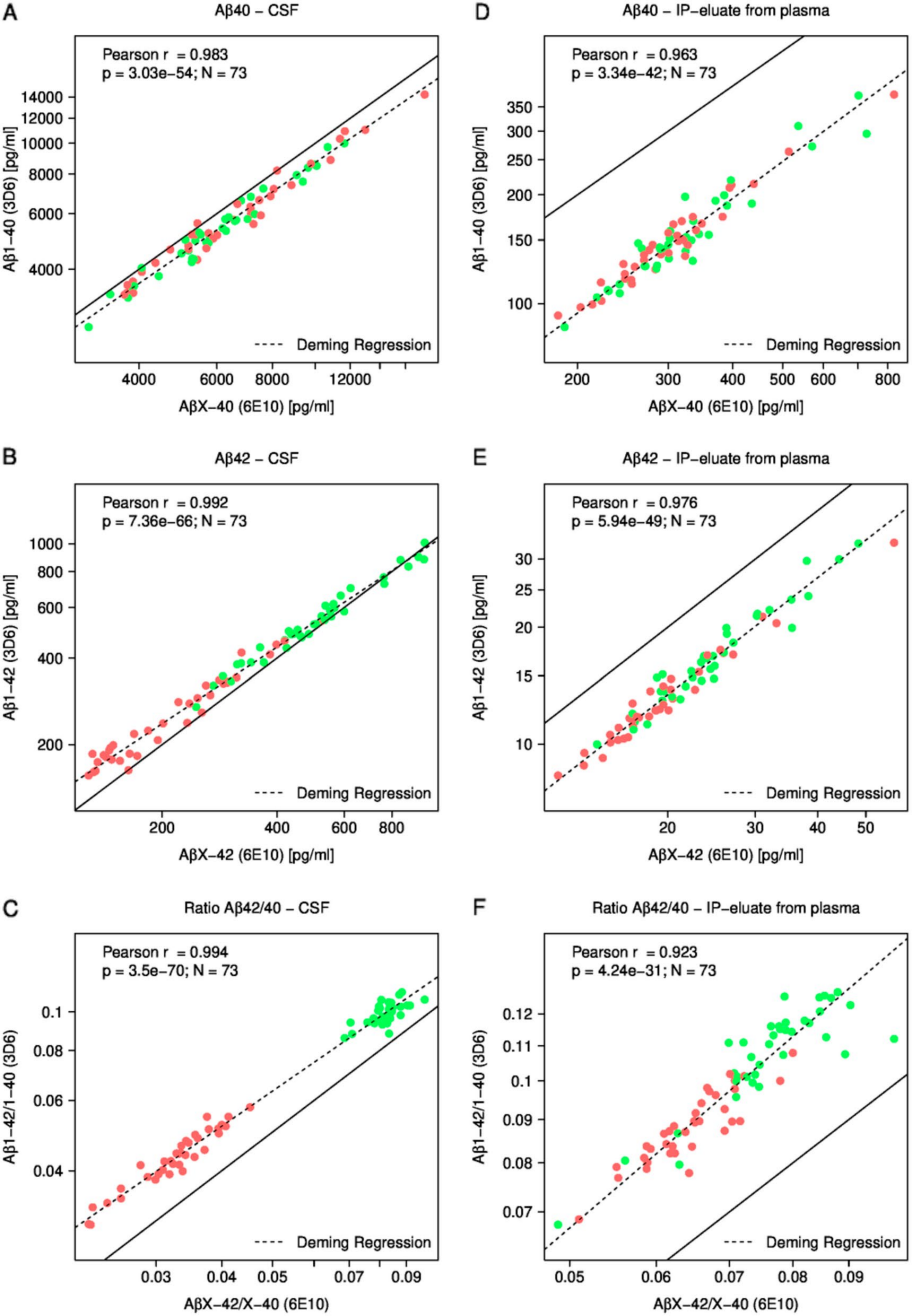
Correlations between Aβ measurements with two different detection antibodies. **A** The CSF Aβ1–40 concentrations are plotted against the CSF AβX–40 concentrations. **B** The CSF Aβ1–42 concentrations are plotted against CSF AβX–42 concentrations. **C** The Aβ1–42/1–40 ratios in CSF are plotted against the AβX–42/X–40 ratios. **D** The Aβ1–40 concentrations in IP-eluates from plasma are plotted against the corresponding AβX–40 concentrations. **E** The Aβ1–42 concentrations in IP-eluates from plasma are plotted against the corresponding AβX–42 concentrations. **F** The Aβ1–42/1–40 ratios are plotted against the corresponding AβX–42/X–40 ratios. The indicated Pearson correlation coefficients (r) and p-values were calculated on log2-transformed data. X- and Y-axes are shown on a logarithmic scale. Solid lines indicate the diagonals (lines of identity) and dashed lines show Deming regressions. Amyloid-positive case are colored in red and amyloid-negative cases in green. *Aβ* amyloid-β, *CSF* cerebrospinal fluid, *IP* immunoprecipitation

The CSF and plasma IP-eluate concentrations of AβX–40, AβX–42 (measured with mAb 6E10), Aβ1–40, Aβ1–42 (measured with mAb 3D6) and the corresponding Aβ42/40 ratios were log2 transformed and analyzed pairwise for Pearson correlations (Fig. 3). The log-transformed CSF concentrations of Aβ1–42 and AβX–42 were strongly correlated (Pearson r = 0.992), and this was also the case for Aβ1–40 vs. AβX–40 (r = 0.983) and the corresponding Aβ42/40 ratios (r = 0.994) (Fig. 3A–C). Strong correlations between the Aβ measurements with mAb 6E10 and mAb 3D6 were also found in the plasma IP-eluates (Fig. 3D–F). On the logarithmic scales that are presented, the Deming regression lines of the plasma values were almost parallel to the diagonal (line of identity) indicating a factor on natural scale, but no offset.

A more comprehensive correlation plot (heatmap) including all possible pairwise correlations in this data set is shown in Additional file 1: Figure S1.

### Aβ isoform levels and Aβ42/40 ratios in amyloid-negative and amyloid-positive subjects

The measured concentrations of the different Aβ isoforms and the calculated Aβ42/40 ratios in CSF and in IP eluates obtained from plasma are summarized in Table 2.

**Table 2:** Amyloid-β isoform levels and Aβ42/40 ratios in amyloid-positive and amyloid-negative subjects

	Variable	Median of Aβ^−^	MAD^a^ of Aβ^−^	Median of Aβ^+^	MAD^a^ of Aβ^+^	P-value^b^
CSF	AβX–40	5777.11	1525.46	6283.72	2095.14	0.51
	AβX–42	500.46	128.56	198.00	84.20	1.04E–13
	AβX–42/X–40	0.083	0.003	0.033	0.005	2.08E–13
	Aβ1–40	5224.52	1361.74	5576.23	1686.43	0.43
	Aβ1–42	523.75	133.87	230.40	82.55	1.20E–13
	Aβ1–42/1–40	0.100	0.006	0.044	0.007	2.08E–13
Plasma IP-eluate	AβX–40	301.93	58.61	300.30	60.42	0.37
	AβX–42	23.67	5.95	18.68	3.24	2.14E–04
	AβX–42/X–40	0.077	0.008	0.065	0.006	3.67E–08
	Aβ1–40	145.67	30.88	142.96	33.32	0.55
	Aβ1–42	15.91	3.96	12.23	2.56	1.76E–04
	Aβ1–42/1–40	0.111	0.013	0.088	0.009	1.77E–08

*Aβ* amyloid-β, *CSF* cerebrospinal fluid, *IP* immunoprecipitation

^a^MAD: median absolute deviation scaled with factor 1.4826

^b^Two-tailed Mann–Whitney test (Wilcoxon rank sum test) p-value for the comparison between the amyloid-negative (Aβ^−^, CSF AβX–42/X–40 > 0.058, n = 37) and amyloid-positive (Aβ^+^, CSF AβX–42/X–40 ≤ 0.058, n = 36) groups. Due to the exploratory character of the study, p-values were not corrected for multiple comparisons

The median plasma IP-eluate concentrations of AβX–42 and Aβ1–42 as well as the AβX–42/X–40 and Aβ1–42/1–40 ratios were statistically significantly lower in the amyloid-positive study participants.

For comparing the AβX–42/X–40 and Aβ1–42/1–40 ratios in plasma and assessing the impact of replacing mAb 6E10 by mAb 3D6 on the detection of amyloid-positivity, we used three different parameters as measures of the magnitude of the effect, (i) the relative median difference, (ii) the relative mean difference and (iii) Cohen’s d (standardized effect size).

**Fig. 4:**
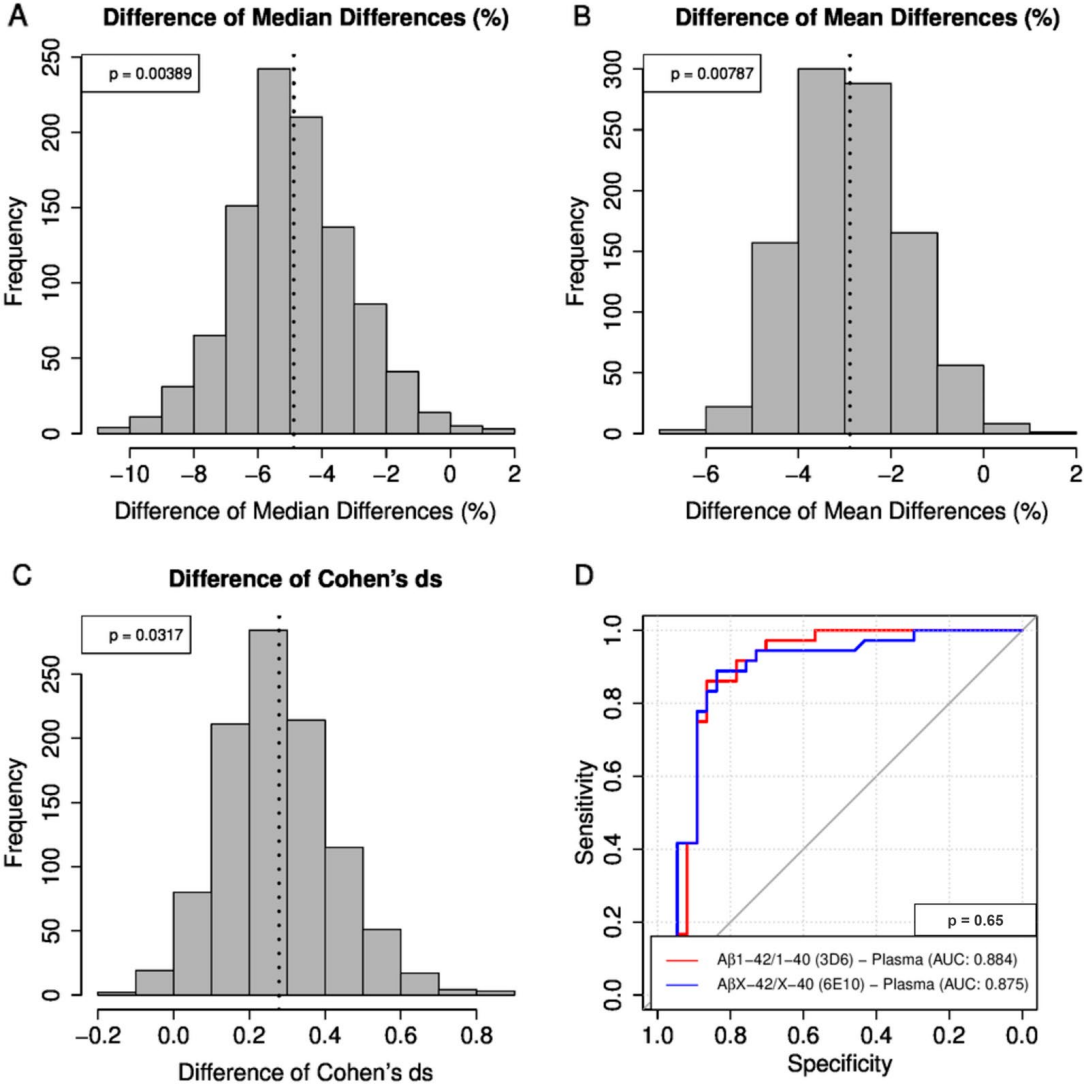
Comparison of plasma AβX–42/X–40 vs. Aβ1–42/1–40 by Bootstrapping statistics and receiver operating characteristic analysis. **A**–**C** histograms of the magnitude of the group differences in plasma AβX–42/X–40 (6E10) vs. Aβ1–42/1–40 (3D6) between amyloid-positive and amyloid-negative subjects observed after .632 bootstrapping are shown. **A** relative median difference, **B** relative mean difference and **C** Cohen’s d as measures of effect size. The indicated p-values were not corrected for multiple comparisons. The dashed lines show the respective means. **D** Receiver operating characteristic (ROC) analysis for plasma Aβ1–42/1–40 and AβX–42/X–40. Single value receiver operating characteristic (ROC) curves for the discrimination between amyloid-positive and amyloid-negative subjects were calculated for the plasma ratios Aβ1–42/1–40 and AβX–42/X–40 measured with mAb 3D6 and 6E10, respectively. The areas under the curves (AUCs) and the p-value are indicated. *Aβ* amyloid-β, *mAb* monoclonal antibody

The median Aβ1–42/1–40 ratio was 20.86% lower in amyloid-positive subjects than in the amyloid-negative group, while the median AβX–42/X–40 ratio was only 15.56% lower. The relative mean difference between amyloid-positive and amyloid-negative subjects was −﻿18.34% for plasma Aβ1–42/1–40 compared to −15.50% for AβX–42/X–40. Cohen’s d, which is a very common measure of effect size, was 1.73 for Aβ1–42/1–40 and 1.48 for plasma AβX–42/X–40. Thus, all three of the tested parameters appeared to indicate an accentuated effect after replacing mAb 6E10 by mAb 3D6 in the Aβ multiplex immunoassay (Additional file 1: Table S1). In order to assess whether the apparent improvement reached statistical significance, we performed .632 bootstrapping (re-sampling from the study participants with replacements). We performed 1000 replications of the .632 bootstrapping and calculated the change in the effect size resulting from measuring Aβ1–42/1–40 instead of AβX–42/X–40. With all three of the tested parameters (relative median difference, relative mean difference and Cohen’s d) unadjusted p-values < 0.05 were obtained (Fig. 4A–C). The ROC curves for the classification of the study participants into the subgroups amyloid-negative and amyloid-positive were very similar with areas under the curves (AUCs) of 0.875 and 0.884, respectively (p = 0.65, DeLong test) (Fig. 4D). A summary of the classification statistics can be found in Additional file 1: Table S2.

## Discussion

In this exploratory study we compared the blood plasma ratios AβX–42/X–40 and Aβ1–42/1–40 for detecting low CSF Aβ42/40 as a surrogate biomarker of the amyloid pathology observed in AD brains. The above-named amino- and carboxy-terminal Aβ variants in plasma were assessed by two-step immunoassays, comprising semi-automated magnetic bead Aβ IP followed by quantification on chemiluminescence multiplex immunoassays. The original commercially available MSD V-Plex Aβ panel 1 (6E10) assay kit employs mAb 6E10 for detecting AβX–40, AβX–42 and AβX–38. The prefix “X” indicates that the assay can detect the canonical Aβ species starting with Asp(1), but also Aβ-variants with shorter or longer N-termini. For specifically measuring Aβ1–40 and Aβ1–42 in the IP eluates, we replaced mAb 6E10 by mAb 3D6, which shows high preference for Aβ variants with a free N-terminal Asp(1) (Fig. 1 and [36]).

We found that the median and mean AβX–42/X–40 plasma ratios (measured with mAb 6E10) were decreased by 15–16% in amyloid-positive study participants relative to amyloid-negative subjects. This figure is in reasonable agreement with published observations [1, 2, 15]. The differences in the corresponding median and mean Aβ1–42/1–40 plasma ratios (measured with mAb 3D6) between the amyloid pathology subgroups were 20.86% and 18.34%, respectively, indicating an accentuation of contrast. The effect sizes (Cohen’s d) were 1.48 for plasma AβX–42/X–40 and 1.73 for Aβ1–42/1–40. For all three parameters (difference in medians, difference in means and Cohen’s d) unadjusted p-values < 0.05 were observed after applying bootstrapping statistics.

It has been estimated that approximately 30–50% of the Aβ peptides in blood originate from the CNS [1]. This estimate was based on earlier stable isotope labeling kinetics studies of Aβ and measurements of arterial-venous differences across the blood–brain barrier (BBB) [37]. Major routes of Aβ clearance from the CNS into venous blood include direct transport across the blood brain barrier and transport into CSF with subsequent reabsorption into the venous blood [37, 38]. While most of the soluble Aβ peptides in CSF seem to carry a free N-terminal aspartic acid [Asp(1)] [17–23], an appreciable fraction of Aβ variants in plasma has a shorter or longer amino terminus [25, 26].

Our finding that the median plasma Aβ1–42/1–40 ratio was decreased by 20.8% in amyloid-positive subjects relative to amyloid-negative individuals while AβX–42/X–40 was decreased by only 15.6% may be explained by a simple model based on the following assumptions:

i. The molecular mechanisms causing the selective decrease in soluble Aβ42 in Alzheimer’s disease are restricted to the CNS and are not mirrored in the particular fraction of soluble plasma Aβ originating from peripheral sources.
ii. The pool of soluble Aβ that is measured in CSF is a reflection of highly soluble Aβ in brain interstitial fluid and contains only negligible amounts of Aβ peptides with a different N-terminus than Asp(1).
iii. N-terminal Aβ variants being measured in plasma and not starting with Asp(1) originate predominantly from the periphery and account for roughly 20–30%. This is a ballpark figure based on published 2D-Western-blot data [25].
iv. Approximately 30% of plasma Aβ originates from the CNS, most of which starting with Asp(1) (see above).
v. Aβ42/40 in CSF (and presumably also in brain interstitial fluid) is approximately 50% lower in amyloid-positive compared to amyloid-negative subjects. The magnitude of the corresponding effect in plasma is substantially smaller due to dilution effects caused by Aβ originating from peripheral sources.

Based on this highly simplified model, we estimate the magnitude of the measurable difference in plasma AβX–42/X–40 between amyloid-negative and amyloid-positive subjects to be approximately 30% of the corresponding difference in CSF AβX–42/X–40. This would suggest a 15% decrease in plasma AβX–42/X–40, which is consistent with published data [1, 2, 15]. Measuring exclusively plasma Aβ1–42 and Aβ1–40 instead of AβX–42 and AβX–40 should increase the relative contribution of CNS-derived Aβ from approximately 30% (see above) to approximately 38%. For example, of a total amount of 100 ng of plasma AβX–40 plus AβX–42 being measured, 70 ng (70%) originate from peripheral sources (see above). Thereof, roughly 30% (approx. 21 ng) do not start with a free N-terminal Asp(1), and are thus not detected if assays specific for a free N-terminal Asp(1) are employed. Consequently, a total amount of only 79 ng of Aβ1–40 plus Aβ1–42 (instead of 100 ng of AβX-40 plus AβX–42) is detected, 30 ng (38%) of which originating from the CNS. In that case, the expected difference in plasma Aβ1–42/1–40 is calculated as 0.38 × 50% = 19%. A graphical illustration of the model can be found in the supplementary information (Additional file 1: Figure S2).

While our findings support the hypothesis that the measurable relative difference in plasma Aβ1–42/1–40 between amyloid-positive and amyloid-negative subgroups is larger than that of AβX–42/X–40, we did not observe a substantial improvement in the AUC in ROC analysis. The study cohort was carefully pre-selected and may thus not be representative of the more heterogeneous population of patients that are seen in a normal clinical setting. Therefore, the immediate relevance of our observations for screening, participant selection for clinical trials or biomarker-supported AD diagnosis in a clinical setting is currently not clear. Nevertheless, our observations may aid, for example, in antibody selection for assay development and optimization.

Strengths of this study include that the measurements of the plasma AβX–42/X–40 and Aβ1–42/1–40 ratios were executed in parallel aliquots of the same IP-eluates and with very closely related multiplex Aβ-immunoassays differing only in the detection antibody in use. All Aβ measurements in CSF and IP-eluates from plasma were performed on the MSD platform in the same laboratory and essentially according to the same routine. Regarding the use for hypothesis testing in this study, the careful selection of the study cohort allowing for a very clear neurochemical dichotomization into subgroups according to the CSF AβX–42/X–40 ratio can be seen as another strength. Limitations of the study include the lack of confirmatory neuropathological data and that the measurements of Aβ concentrations in IP-eluates from blood plasma have to be considered relative and do not allow for translation into absolute values. However, this we consider less compromising when ratios are used for assessment. Further limitations are the rather small number of subjects included in this idealized sample and the use of mAb 1E8 for immunoprecipitation. This monoclonal antibody is directed against an amino-terminal epitope within the Aβ peptide and can detect only a subset of amino-terminally truncated Aβ-variants that may potentially be present in human blood.

Further studies should address whether our findings can be confirmed in an independent and larger sample and possibly using additional, preferably automated assay platforms.

## Conclusions

Our findings support the hypothesis that the measurable decrease in plasma Aβ42/40 in the presence of cerebral amyloid pathology can be accentuated to some extent by employing assays that specifically measure Aβ peptides carrying a free N-terminal aspartic acid residue [Asp(1)]. The observations may aid in assay development and optimization.

## Supplementary Information

**Additional file 1: Figure S1.** Analysis of correlations between Aβ measures in cerebrospinal fluid and eluates obtained after immunoprecipitation from EDTA-blood plasma. Pairwise correlation analysis of the Aβ measures in cerebrospinal fluid and plasma. The heatmap shows Pearson correlation coefficients between Aβ-variants and Aβ42/40 ratios measured in cerebrospinal fluid (CSF) and in blood plasma. Pearson correlation coefficients were calculated on log2 transformed values (except for ratios). Cluster dendrograms (complete linkage clustering) are shown on top and on left hand side. **Figure S2.** Hypothetical model to explain the observed enhancement of the differences between amyloid-positive and amyloid-negative patients in plasma Aβ42/40 by measuring exclusively plasma Aβ1–42 and Aβ1–40. According to our model, the molecular mechanisms causing the selective, approximately 50% reduction in CSF Aβ42/40 in the presence of brain amyloid (Keshavan, Wellington et al. 2021) are restricted to the CNS. We assume that approximately 30% (in this example 30 ng of a measured total amount of 100 ng) of soluble Aβ in blood plasma originates from the central nervous system (CNS), most of which starting with Asp(1). Of the remaining plasma Aβ originating from peripheral sources, approximately 30% is estimated to have a different N-terminus. The monoclonal antibody (mAb) 6E10 detects several aminoterminal Aβ variants (i.e. AβX–40 and AβX–42). The measurable decrease in plasma Aβ42/40 in amyloid-positive patients is proportional to the fraction of plasma Aβ in the assay that originates from the CNS. Measuring exclusively Aβ1–40 and Aβ1–42 (instead of AβX–40 and AβX–42) by employing mAb 3D6 will increase the relative fraction of Aβ originating from CNS from 30% (when measured with mAb 6E10) to 38% because Aβ peptides with other N-termini than Asp(1) are excluded from the measurements with mAb 3D6. In consequence, the measurable decrease in plasma Aβ1–42/1–40 in amyloid-positive subjects is expected to be larger than that of AβX–42/X–40. The assumed 50% reduction in CSF Aβ42/40 in the presence of brain amyloid is expected to be mirrored in plasma by a 15﻿% (0.3 × 50%) reduction in AβX–42/X–40 but 19% decrease (0.38 × 50%) in Aβ1–42/1–40. **Table S1.** Comparison of group differences: Plasma AβX–42/X–40 vs. Aβ1–42/Aβ1–40. **Table S2.** Classification statistics for detection of amyloid-positivity for plasma Aβ1–42/1–40 and AβX–42/X–40.

## Data Availability

The datasets used and/or analyzed in the present study are available from the corresponding author on reasonable request.

## Abbreviations

Aβ: Amyloid-β
AD: Alzheimer’s disease
AUC: Area under the curve
CIEF immunoassay: Capillary isoelectric focusing immunoassay
CNS: Central nervous system
CSF: Cerebrospinal fluid
IP: Immunoprecipitation
IP-MS: Immunoprecipitation followed by mass spectrometry
mAb: Monoclonal antibody
MAD: Median absolute deviation scaled with factor 1.4826
MALDI-TOF-MS: Matrix-assisted laser desorption/ionization time-of-flight mass spectrometry
MSD: Mesoscale discovery
ROC analysis: Receiver operating characteristic analysis
