# Supplementary material for: Is plasma amyloid-β 1–42/1–40 a better biomarker for Alzheimer’s disease than AβX–42/X–40?
Source: Fluids Barriers CNS. 2022 Dec 3;19:96. doi: 10.1186/s12987-022-00390-4 (PMC9719149; doi:10.1186/s12987-022-00390-4)
Supplement: Supplementary file 1 — Additional file 1: Figure S1. Analysis of correlations between Aβ measures in cerebrospinal fluid and eluates obtained after immunoprecipitation from EDTA-blood plasma. Pairwise correlation analysis of the Aβ measures in cerebrospinal fluid and plasma. The heatmap shows Pearson correlation coefficients between Aβ-variants and Aβ42/40 ratios measured in cerebrospinal fluid (CSF) and in blood plasma. Pearson correlation coefficients were calculated on log2 transformed values (except for ratios). Cluster dendrograms (complete linkage clustering) are shown on top and on left hand side. Figure S2. Hypothetical model to explain the observed enhancement of the differences between amyloid-positive and amyloid-negative patients in plasma Aβ42/40 by measuring exclusively plasma Aβ1–42 and Aβ1–40. According to our model, the molecular mechanisms causing the selective, approximately 50% reduction in CSF Aβ42/40 in the presence of brain amyloid (Keshavan, Wellington et al. 2021) are restricted to the CNS. We assume that approximately 30% (in this example 30 ng of a measured total amount of 100 ng) of soluble Aβ in blood plasma originates from the central nervous system (CNS), most of which starting with Asp(1). Of the remaining plasma Aβ originating from peripheral sources, approximately 30% is estimated to have a different N-terminus. The monoclonal antibody (mAb) 6E10 detects several aminoterminal Aβ variants (i.e. AβX–40 and AβX–42). The measurable decrease in plasma Aβ42/40 in amyloid-positive patients is proportional to the fraction of plasma Aβ in the assay that originates from the CNS. Measuring exclusively Aβ1–40 and Aβ1–42 (instead of AβX–40 and AβX–42) by employing mAb 3D6 will increase the relative fraction of Aβ originating from CNS from 30% (when measured with mAb 6E10) to 38% because Aβ peptides with other N-termini than Asp(1) are excluded from the measurements with mAb 3D6. In consequence, the measurable decrease in plasma Aβ1–42/1–40 in amyloid-positive subjects [file 12987_2022_390_MOESM1_ESM.pdf]

Supplementary material

Is Plasma Amyloid-β 1–42/1-40 a Better Biomarker for Alzheimer's Disease than AβX–42/X–40?

Hans-Wolfgang Klafki[a], Barbara Morgado[a], Oliver Wirths[a], Olaf Jahn[a, b], Chris Bauer[c], Hermann Esselmann[a], Johannes Schuchhardt[c], Jens Wiltfang[a, d, e]

[a]Department of Psychiatry and Psychotherapy, University Medical Center Goettingen, Georg-August-University, Goettingen, Germany
[b]Neuroproteomics Group, Department of Molecular Neurobiology, Max Planck Institute for Multidisciplinary Sciences, Goettingen, Germany
[c]MicroDiscovery GmbH, Marienburger Strasse 1, D-10405 Berlin, Germany
[d]German Center for Neurodegenerative Diseases (DZNE), Goettingen, Germany
[e]Neurosciences and Signaling Group, Institute of Biomedicine (iBiMED), Department of Medical Sciences, University of Aveiro, Aveiro, Portugal

**Correspondence to Hans-W. Klafki, Dept. of Psychiatry and Psychotherapy, University Medical Center Goettingen, Georg-August-University, Von-Siebold-Str. 5, D37075 Goettingen, Germany; e-mail: hans.klafki@med.uni-goettingen.de, tel.: +49 5513965797*

Content:

Additional file 1: Figure S1. Analysis of correlations between Aβ measures in cerebrospinal fluid and eluates obtained after immunoprecipitation from EDTA-blood plasma.

Additional file 1: Figure S2. Hypothetical model to explain the observed enhancement of the differences between amyloid-positive and amyloid-negative patients in plasma Aβ42/40 by measuring exclusively plasma Aβ1–42 and Aβ1–40.

Additional file 1: Table S1. Comparison of group differences: Plasma AβX–42/X–40 vs. Aβ1–42/Aβ1–40.

Additional file 1: Table S2. Classification statistics for detection of amyloid-positivity for plasma Aβ1–42/1–40 and AβX–42/X–40.

Additional file 1: Figure S1. Analysis of correlations between Aβ measures in cerebrospinal fluid and eluates obtained after immunoprecipitation from EDTA-blood plasma.

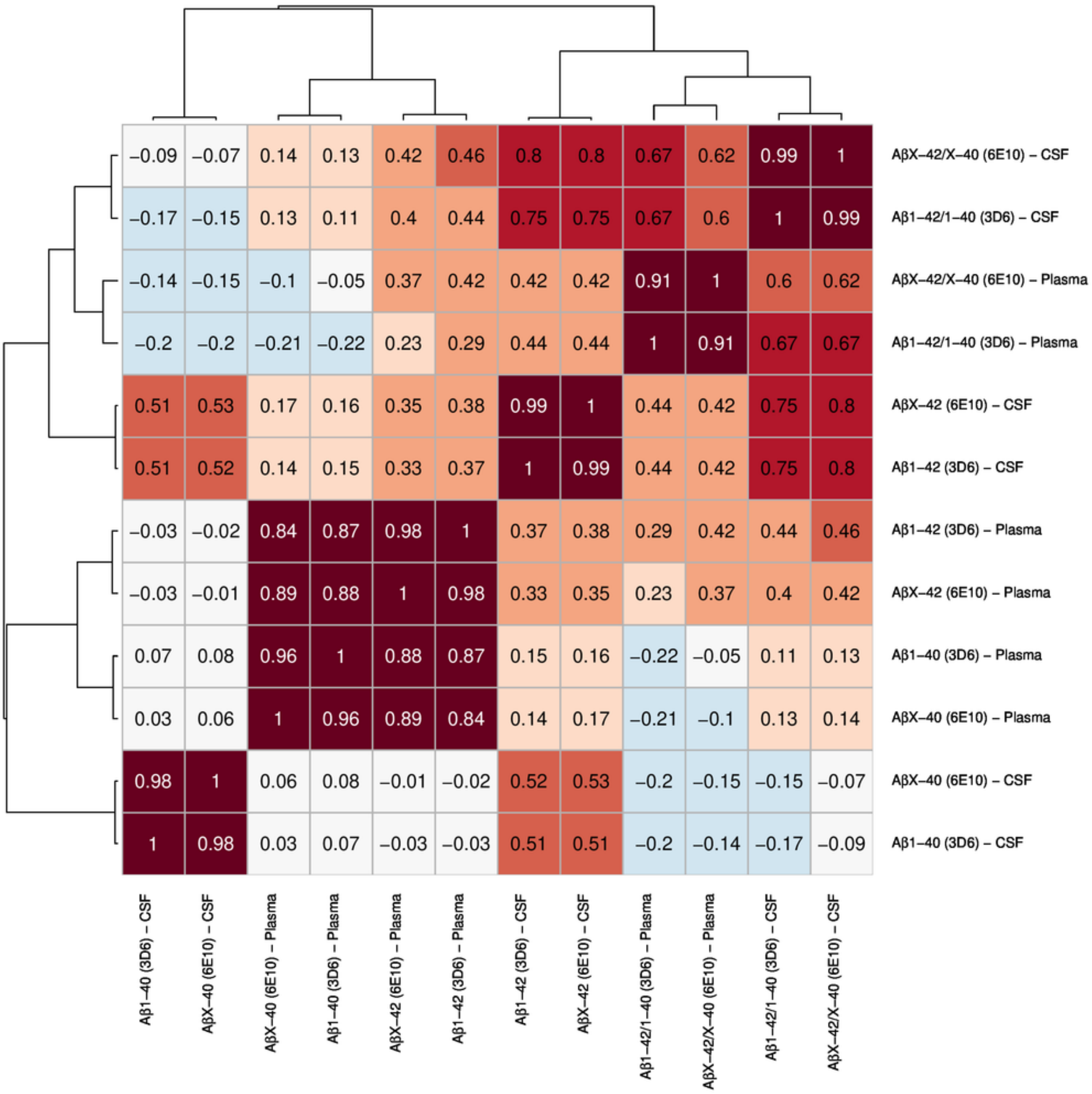

Additional file 1: Figure S1. Analysis of correlations between Aβ measures in cerebrospinal fluid and eluates obtained after immunoprecipitation from EDTA-blood plasma. Pairwise correlation analysis of the Aβ measures in cerebrospinal fluid and plasma. The heatmap shows Pearson correlation coefficients between Aβ-variants and Aβ42/40 ratios measured in cerebrospinal fluid (CSF) and in blood plasma. Pearson correlation coefficients were calculated on log2 transformed values (except for ratios). Cluster dendrograms (complete linkage clustering) are shown on top and on left hand side.

Additional file 1: Figure S2. Hypothetical model to explain the observed enhancement of the differences between amyloid-positive and amyloid-negative patients in plasma Aβ42/40 by measuring exclusively plasma Aβ1–42 and Aβ1–40.

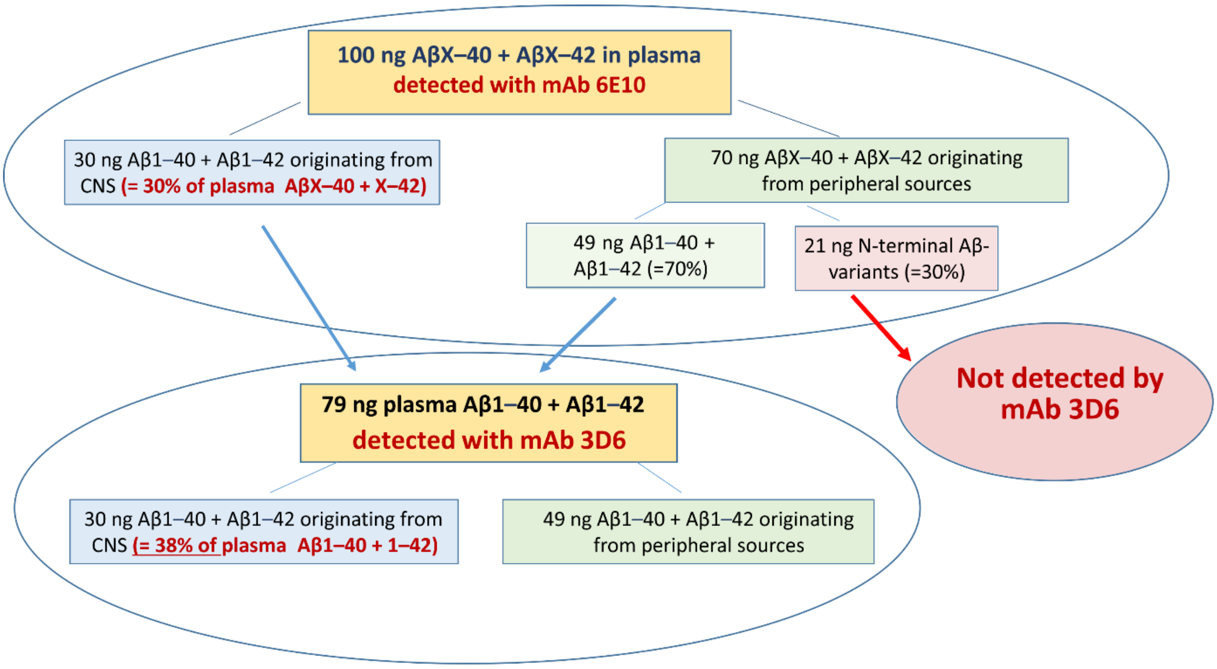

Additional file 1: Figure S2: Hypothetical model to explain the observed enhancement of the differences between amyloid-positive and amyloid-negative patients in plasma Aβ42/40 by measuring exclusively plasma Aβ1–42 and Aβ1–40. According to our model, the molecular mechanisms causing the selective, approximately 50% reduction in CSF Aβ42/40 in the presence of brain amyloid (Keshavan, Wellington et al. 2021) are restricted to the CNS. We assume that approximately 30% (in this example 30 ng of a measured total amount of 100 ng) of soluble Aβ in blood plasma originates from the central nervous system (CNS), most of which starting with Asp(1). Of the remaining plasma Aβ originating from peripheral sources, approximately 30% is estimated to have a different N-terminus. The monoclonal antibody (mAb) 6E10 detects several aminoterminal Aβ variants (i.e. AβX–40 and AβX–42). The measurable decrease in plasma Aβ42/40 in amyloid-positive patients is proportional to the fraction of plasma Aβ in the assay that originates from the CNS. Measuring exclusively Aβ1–40 and Aβ1–42 (instead of AβX–40 and AβX–42) by employing mAb 3D6 will increase the relative fraction of Aβ originating from CNS from 30% (when measured with mAb 6E10) to 38% because Aβ peptides with other N-termini than Asp(1) are excluded from the measurements with mAb 3D6. In consequence, the measurable decrease in plasma Aβ1–42/1–40 in amyloid-positive subjects is expected to be larger than that of AβX–42/X–40. The assumed 50% reduction in CSF Aβ42/40

in the presence of brain amyloid is expected to be mirrored in plasma by a 15% (0.3 x 50%) reduction in AβX–42/X–40 but 19% decrease (0.38 x 50%) in Aβ1–42/1–40.

Reference:

Keshavan, A., H. Wellington, Z. Chen, A. Khatun, M. Chapman, M. Hart, D. M. Cash, W. Coath, T. D. Parker, S. M. Buchanan, S. E. Keuss, M. J. Harris, H. Murray-Smith, A. Heslegrave, N. C. Fox, H. Zetterberg and J. M. Schott (2021). "Concordance of CSF measures of Alzheimer's pathology with amyloid PET status in a preclinical cohort: A comparison of Lumipulse and established immunoassays." Alzheimers Dement (Amst) **13**(1): e12131.

Additional file 1: Table S1. Comparison of group differences: Plasma AβX–42/X–40 vs. Aβ1–42/Aβ1–40.

Variable	Median difference (%)[1]	Mean difference (%)[2]	Cohen's d[3]
Plasma AβX–42/X–40	−15.56	−15.50	1.48
Plasma Aβ1–42/1–40	−20.86	−18.34	1.73

[1] The relative median difference between amyloid-positive (Aβ+) and amyloid negative (Aβ−) groups was calculated as:

$$Median\ difference\ (\%) = 100 * \frac{median\ (A\beta +) - median\ (A\beta -)}{median(A\beta -)}$$

[2] The relative mean difference between amyloid-positive (Aβ+) and amyloid-negative (Aβ−) groups was calculated as:

$$Mean\ difference\ (\%) = 100 * \frac{mean\ (A\beta +) - mean\ (A\beta -)}{mean(A\beta -)}$$

[3] Cohen's d was calculated with R package "effsize" (version 0.8.1).

Aβ, amyloid-β.

Additional file 1: Table S2. Classification statistics for detection of amyloid-positivity[1] for plasma Aβ1–42/1–40 and AβX–42/X–40.

	Plasma Aβ1–42/1–40	Plasma AβX–42/X–40
True positive	31	32
True negative	32	31
False positive	5	6
False negative	5	4
Positive predictive value	0.861	0.842
Negative predictive value	0.865	0.886
Sensitivity	0.861	0.889
Specificity	0.865	0.838
Accuracy	0.863	0.863
Area under the ROC curve (AUC)	0.884	0.875

Receiver operating characteristic (ROC) curves were evaluated at the maximum Youden point.

[1] The study participants were categorized according to the CSF AβX–42/AβX–40 ratio. Amyloid-β-positive (Aβ+): CSF AβX–42/X–40 ≤ 0.058; amyloid-β-negative (Aβ−): CSF AβX–42/X–40 > 0.058

Aβ, amyloid-β.
